# Health service and medication costs associated with common mental disorders and subthreshold symptoms in women: Findings from the Geelong Osteoporosis Study in Australia

**DOI:** 10.1177/00048674241229931

**Published:** 2024-02-11

**Authors:** Mary Lou Chatterton, Jan Faller, Long Khanh-Dao Le, Lidia Engel, Lana J Williams, Julie A Pasco, Cathy Mihalopoulos

**Affiliations:** 1 School of Public Health and Preventive Medicine, Monash University, Melbourne, VIC, Australia; 2 Institute for Health Transformation, Deakin University, Geelong, VIC, Australia; 3 Institute for Mental and Physical Health and Clinical Translation (IMPACT), School of Medicine, Barwon Health, Deakin University, Geelong, VIC, Australia; 4 Department of Medicine at Western Health, The University of Melbourne, St Albans, VIC, Australia

**Keywords:** Cost-of-illness, mood disorders, anxiety disorders, subthreshold psychological symptoms

## Abstract

**Objective:**

This analysis estimated 2013 annual healthcare costs associated with the common mental disorders of mood and anxiety disorders and psychological symptoms within a representative sample of Australian women.

**Methods:**

Data from the 15-year follow-up of women in the Geelong Osteoporosis Study were linked to 12-month Medicare Benefits Schedule and Pharmaceutical Benefits Scheme data. A Structured Clinical Interview for Diagnostic and Statistical Manual of Mental Disorders, Fourth Edition, Non-patient edition identified common mental disorders and the General Health Questionnaire 12 assessed psychological symptoms. Participants were categorised into mutually exclusive groups: (1) common mental disorder (past 12 months), (2) subthreshold (no common mental disorder and General Health Questionnaire 12 score ⩾4) or (3) no common mental disorder and General Health Questionnaire 12 score <4. Two-part and hurdle models estimated differences in service use, and adjusted generalised linear models estimated mean differences in costs between groups.

**Results:**

Compared to no common mental disorder, women with common mental disorders utilised more Medicare Benefits Schedule services (mean 26.9 vs 20.0, *p* < 0.001), had higher total Medicare Benefits Schedule cost ($1889 vs $1305, *p* < 0.01), received more Pharmaceutical Benefits Scheme prescriptions (35.8 vs 20.6, *p* < 0.001), had higher total Pharmaceutical Benefits Scheme cost ($1226 vs $740, *p* < 0.05) and had significantly higher annual out-of-pocket costs for Pharmaceutical Benefits Scheme prescriptions ($249 vs $162, *p* < 0.001). Compared to no common mental disorder, subthreshold women were less likely to use any Medicare Benefits Schedule service (89.6% vs 97.0%, *p* < 0.01), but more likely to use mental health services (11.4% vs 2.9%, *p* < 0.01). The subthreshold group received more Pharmaceutical Benefits Scheme prescriptions (mean 43.3 vs 20.6, *p* < 0.001) and incurred higher total Pharmaceutical Benefits Scheme cost ($1268 vs $740, *p* < .05) compared to no common mental disorder.

**Conclusions:**

Common mental disorders and subthreshold psychological symptoms place a substantial economic burden on Australian healthcare services and consumers.

## Introduction

The most recent Australian cost-of-illness estimates for common mental disorders (CMDs) in adults, using the 2007 National Survey of Mental Health and Wellbeing (NSMHWB), estimated total annual health care costs of approximately $974 million and lost productivity costs of $11,800 million (Lee et al., 2017). Anxiety disorders were the single largest contributor to total annual health care costs ($376 million) and lost productivity costs ($4990 million), due to the high prevalence of these disorders (over 50%). However, average annual health care costs and average annual number of lost working days were highest for people diagnosed with multiple CMDs. These results, while useful in estimating the economic burden of CMDs, were calculated by applying standard unit costs to self-reported health care resource use and productivity loss data subject to recall bias.

Cost-of-illness estimates are highly dependent on the prevalence of a condition. Global and national data indicate that women are more likely to meet criteria for depression, anxiety or eating disorders relative to men (Australian Bureau of Statistics, 2008; Ferrari et al., 2022). The COVID-19 pandemic exacerbated this disparity, with an increase in CMD diagnoses for women compared to men, largely driven by higher prevalence rates of depression and anxiety in women (Australian Bureau of Statistics, 2022; Santomauro et al., 2021). Women with CMDs are also more likely to see a health professional and use digital services for their mental health compared to men contributing to overall healthcare costs (Australian Bureau of Statistics, 2022).

Across the spectrum of mental disorders (MDs), life expectancy is substantially reduced in comparison to the general population due to a combination of poor physical health, related to comorbid non-communicable diseases, and suicide (Lawrence et al., 2013; Thornicroft, 2013). The risk of cardiometabolic disease (hypertension, stroke, diabetes, metabolic syndrome, obesity) is increased 1.4 to 2.0 times for individuals with MDs compared to the general population (Firth et al., 2019). However, few studies have reported the cost of physical comorbidity with MDs and these have been limited to the United States (Bryant-Comstock et al., 2002; Centorrino et al., 2009; Greenberg et al., 2015, 2021).

Another under researched area is the cost for individuals with mental health symptoms that do not reach diagnostic thresholds. Previous analysis of the Young Minds Matter survey linked with resource use and cost from the Australian Medicare Benefits Schedule (MBS) and Pharmaceutical Benefits Scheme (PBS) identified that young people (ages 4–17 years) with symptoms but without a diagnosis had significantly greater medical service and medication costs compared to a cohort without symptoms or diagnoses (Le et al., 2021). The additional burden and cost associated with subthreshold MD have not been evaluated in adults.

This study aimed to estimate the direct annual non-hospital healthcare costs associated with the CMDs of mood and anxiety disorders and subthreshold psychological symptoms within a representative sample of Australian women residing in regional Victoria during 2013. The analysis was undertaken using a health sector perspective.

## Methods

### Participants

The Geelong Osteoporosis Study (GOS) is a population-based study originally designed to investigate the epidemiology of osteoporosis in Australia (Pasco et al., 2012). Subsequent GOS assessment phases included a broader range of health measures and an assessment of non-communicable diseases including CMDs. Participants were recruited from electoral rolls for the Barwon Statistical Division, a geographic area surrounding the regional city of Geelong in the state of Victoria, Australia.

The GOS enrolled and followed up both men and women through separate waves of data collection with the 15-year follow-up for women completed in 2013 and was used in this secondary analysis. The 15-year follow-up for male participants was not available at the time of this analysis.

### Research ethics and patient consent

The GOS and this analysis were approved by the Human Research Ethics Committees at Barwon Health (Geelong, Victoria, Australia, Project ID 92/01) and Deakin University (Geelong, Victoria, Australia). All participants provided written informed consent to participate in the study, and separate written consent for access to MBS and PBS data.

### MD classification

The Structured Clinical Interview for Diagnostic and Statistical Manual of Mental Disorders, Fourth Edition, Non-patient edition (SCID-I/NP) was utilised to assess lifetime history of CMDs for all GOS participants including mood disorder (major depressive disorder, minor depressive disorder, dysthymia, mood disorder due to a general medical condition [GMC], substance-induced mood disorders and bipolar disorder I, II and not otherwise specified [NOS]), anxiety disorder (panic disorder, agoraphobia, social phobia, specific phobia, obsessive-compulsive disorder, generalised anxiety disorder, anxiety disorders due to GMC, substance-induced anxiety disorder and anxiety disorders [NOS]) and substance use (abuse and dependence) and eating disorders (anorexia/bulimia nervosa and binge eating disorder). The SCID-I/NP was administered by personnel with psychology qualifications (Williams et al., 2010). For this analysis, participants with a single diagnosis of a substance use or eating disorder were excluded. The remaining participants were classified as having met criteria for any mood or anxiety disorder in the past 12 months (‘CMD’) or having no history of a CMD (‘no-CMD’). Participants were also placed into subgroups based on the presence of a single type of 12-month CMD diagnosis (only anxiety diagnoses, only mood diagnoses) or multiple diagnoses (‘multiple-CMD’) which included combinations of anxiety, mood and other diagnoses.

### Assessment of subthreshold psychological symptoms

Participants were classified into a subthreshold group based on their responses to the General Health Questionnaire 12 (GHQ-12), a commonly used self-report screening questionnaire to detect non-psychotic psychiatric disorders (Goldberg, 1978). Participants with a score equal to or above the threshold of four without a current CMD according to the SCID-I/NP were identified as ‘subthreshold’ (Sanna et al., 2013).

### Demographic data

Demographic information including age, marital status, educational attainment and employment status was self-reported by study participants through a questionnaire.

### Healthcare service use and cost

Administrative data from MBS and PBS provided details of each service utilised by participants covering different time periods, but were trimmed to correspond to the 12 months preceding the SCID-I/NP date for each participant. Participants were encouraged to provide access to both MBS and PBS data, although they could consent to provide access to only one set of records. MBS services are subsidised by the Australian Government and mainly include community-based healthcare (outside of public hospitals), although some services utilised during private hospital admissions are billed through MBS. Some services are fully covered by the Australian Government at the full benefit, and some are partially covered. Service providers are able to charge fees above the MBS reimbursed amount. The difference in the actual fee and benefit paid is covered by the patient as an out-of-pocket (OOP) cost (Department of Health, 2012a).

The PBS is a comprehensive compendium of prescription medications subsidised by the Australian Government. The cost of listed medications is shared by the government and the patient through a co-payment system (Department of Health, 2012b).

The administrative data included the specific service used by date together with the documented amounts paid by the government and the patient. Costs are presented in 2013 Australian dollars ($) since this was the latest year of data. MBS and PBS costs not in 2013 dollars were converted using the Total Health Price Index (Australian Institute of Health and Welfare, 2019).

#### General and MD-related MBS services

Diagnostic information is not available with MBS data, hence it is not known with certainty whether a particular service was related to a specific diagnosis. However, the Better Access (BA) initiative provides rebates for mental health services, and uses specific MBS item numbers to reimburse for general practitioner (GP), specialist, psychologist and some allied health services (Department of Health and Aged Care, 2019). BA requires a referral from a medical practitioner allowing reimbursement for up to 10 individual and 10 group allied mental health services per calendar year. To make the distinction for this study, MD services were defined as mental health plans and psychological therapies or services provided by a psychiatrist, psychologist, mental health nurse, social worker or a mental health occupational therapist using BA-specific MBS item numbers. All other MBS services were classified as general health related.

#### General and MD-related PBS prescriptions

The PBS data do not include diagnostic information. Classification of MD and general medications was based on the Anatomical Therapeutic Chemical Classification System (WHO Collaborating Centre for Drug Statistics Methodology, 2018). Medications used in the treatment of MD were identified as antipsychotics (N05A), anxiolytics (N05B), hypnotics and sedatives (N05C), antidepressants (N06A) or psychostimulants and nootropics (N06B). We also included classification N03A (anticonvulsants) specifically carbamazepine, clonazepam, oxcarbazepine, pregabalin, sodium valproate and topiramate under MD medications given these medications are commonly used in MD management. All other medications were classified for general health.

### Statistical analysis

Women participating in the GOS 15-year follow-up who underwent a SCID-I/NP, completed a GHQ-12 as well as consented to use of MBS or PBS data were included in the analyses.

Statistical analyses were conducted in StataSE 16 (StataCorp, 2017). Descriptive statistics by MD and general MBS and PBS service costs were analysed using mean and 95% confidence intervals.

Two-part models were used to estimate whether there was a difference in the probability of incurring any costs (first part) and the difference in costs (second part) of those who used services. The first part was a logistic regression model for the probability of incurring any costs and the second part was a generalised linear model (GLM) for costs incurred conditional that the cost is greater than zero (Deb and Norton, 2018).

The GLMs adjusted for age, marital status, educational attainment and employment status were used to estimate the mean differences in costs (dependent variable) between classification groups (independent variable: CMD, no-CMD, subthreshold, etc.). A gamma error distribution with a logarithmic link function in the GLM was used due to the skewed distribution of cost data consistent with the International Society for Pharmacoeconomics and Outcome Research guidelines (Ramsey et al., 2015).

A hurdle model was used to detect differences between groups in the number of healthcare services. A hurdle model is the equivalent of a two-part model but combines a binary model to predict zeros with a zero-truncated negative binomial model to predict non-zero counts (Deb and Norton, 2018).

## Results

**Figure 1.**
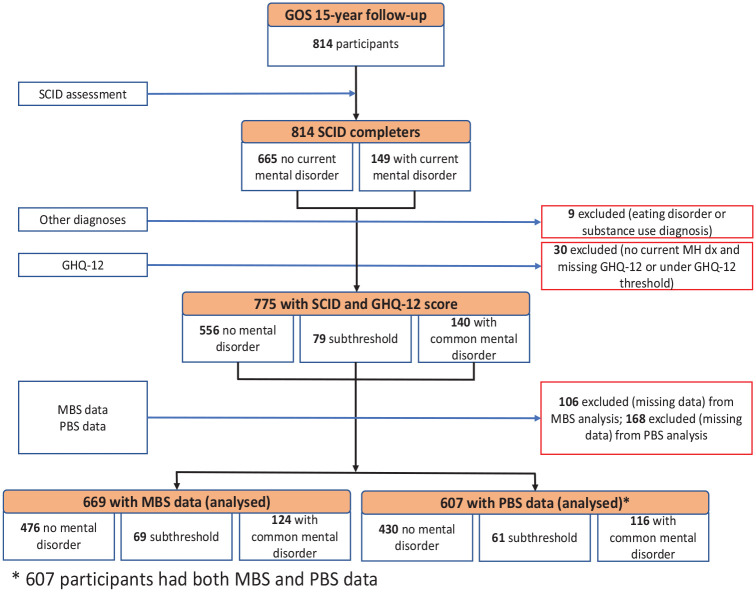
Flow diagram for data selection process. *A total of 607 participants had both MBS and PBS data.

Figure 1 presents the process of selecting the final dataset for analysis. Of 814 women who participated in the 15-year follow-up, those for whom SCID-I/NP or GHQ-12 data were not available were excluded (*n* = 39), resulting in 775 participants (95%) for potential inclusion in this study. An additional 106 and 168 participants did not have MBS and PBS data available, respectively, and were therefore excluded from the analyses resulting in 607 (75%) participants having both MBS and PBS data.

The mean age of respondents was 55.7 years (range: 28–94 years). There were 556 (72%) participants with no history of a CMD or current psychological symptoms, 79 (10%) had current psychological symptoms and the remaining 140 (18%) met criteria for a 12-month CMD. Of the 12-month CMD group (*n* = 140), 60 (43%) met criteria for an anxiety disorder and 30 (21%) met criteria for a mood disorder. A total of 50 (36%) participants had at least two co-occurring 12-month MDs.

### Service use and costs to MBS

**Figure 2.**
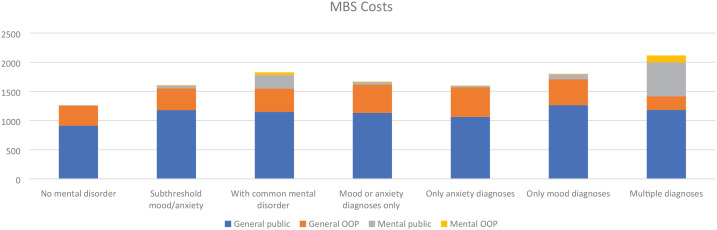
Average annual Medicare Benefits Schedule cost by mental disorder classification. ^a^Includes participants with a combination of at least two categories of diagnosis from anxiety, mood, and other (comorbid eating or substance use disorders).

Figure 2 shows the average annual MBS cost by CMD classification. There were 669 participants with MBS data (86% of all participants). Of these, 22 (3%) did not use any MBS services and therefore had zero MBS costs. Descriptive analysis shows that both subthreshold and CMD groups were associated with higher average annual MBS costs than the no-CMD group. The average MBS costs incurred were $1263 for no-CMD, $1608 for subthreshold and $1828 for the CMD group.

Table 1 presents two-part and hurdle model results for MBS services. Women with subthreshold psychological symptoms were almost four times more likely to use mental health services compared to the no-CMD group (11.4% vs 2.9%, *p* < 0.01). Women with CMD were almost seven times more likely to utilise MBS mental health services compared to the no-CMD group (19.7% vs 2.9%, *p* < 0.001). In particular, women with a mood disorder were eight times more likely to use mental health services (23.4% vs 2.9%, *p* < 0.001), and multiple-MD were 11 times more likely to use mental health services compared to the no-CMD group (32.8% vs 2.9%, *p* < 0.001).

**Table 1.** Two-part and hurdle model results for Medicare Benefits Schedule services in the year preceding mental health assessment.

Disorder classification	Mental health services			General health services			Combined services		
	% of participants using health service % [95% CI]	Annual number of services for those who accessed *N* [95% CI]	Average annual cost for those who accessed services^a^ $ [95% CI]	% of participants using health service % [95% CI]	Annual number of services for those who accessed *N* [95% CI]	Average annual cost for those who accessed services^a^ $ [95% CI]	% of participants using health service % [95% CI]	Annual number of services for those who accessed *N* [95% CI]	Average annual cost for those who accessed services^a^ $ (95% CI]
No common mental disorder	2.9 [1.3, 4.5]	3.4 [1.3, 5.4]	419 [72, 766]	97.0 [95.5, 98.6]	19.9 [18.4, 21.4]	1293 [1133, 1453]	97.0 [95.5, 98.6]	20.0 [18.5, 21.5]	1305 [1142, 1467]
Subthreshold symptoms	11.4 [4.3, 18.6]**	4.1 [1.0, 7.1]	526 [9, 1044]	89.6 [82.4, 96.9]**	27.1 [21.6, 32.5]**	1686 [1124, 2247]	89.6 [82.4, 96.9]**	27.6 [22.0, 33.1]**	1752 [1166, 2337]
With common mental disorder	19.7 [13.0, 26.5]***	8.1 [5.2, 11]*	1152 [568, 1736]*	99.2 [97.5, 100.8]	24.9 [21.2, 28.5]**	1601 [1220, 1983]	99.2 [97.5, 100.8]	26.9 [22.9, 30.8]***	1889 [1434, 2345]**
Mood or anxiety diagnoses only	12.8 [5.9, 19.7]***	3.0 [1.3, 4.7]	303 [95, 511]	98.7 [96.3, 101.2]	24.8 [20.3, 29.3]*	1675 [1184, 2166]	98.7 [96.3, 101.2]	25.3 [20.7, 29.8]*	1724 [1215, 2233]
Only anxiety diagnoses	7.7 [1.0, 14.4]	3.2 [0.2, 6.3]	291 [0, 639]	98.1 [94.3, 101.8]	25.8 [20.0, 31.6]*	1755 [1113, 2397]	98.1 [94.3, 101.8]	26.1 [20.3, 31.9]*	1772 [1119, 2424]
Only mood diagnoses	23.4 [7.9, 39.0]***	2.8 [0.7, 4.9]	309 [46, 572]	100 [NR]^b^	22.9 [16, 29.9]	1529 [772, 2287]	100 [NR]^b^	23.8 [16.6, 31.1]	1638 [820, 2455]
Multiple diagnoses	32.8 [19.3, 46.4]***	11.8 [6.9, 16.7]***	1773 [765, 2781]**	100 [NR]^b^	25.0 [19.0, 31.1]	1469 [889, 2048]	100 [NR]^b^	29.9 [22.7, 37.2]**	2205 [1317, 3092]*

CI: confidence interval; NR: not reportable.

a Cost includes the payments made by the Commonwealth government through the MBS and the out-of-pocket cost made by the consumer.

b Groups without zero values are not estimable or reportable for two-part models.

* *p*<0.05; **p<0.01; ***p<0.001.

Women with CMD who accessed MBS mental health services used about four and a half more services than women with no-MD (8.1 vs 3.4, *p* < 0.05). This translates to an additional $733 in healthcare costs ($1152 vs $419, *p* < 0.05). More specifically, this is driven by multiple-MD utilising eight more services on average than no-CMD (11.8 vs 3.4, *p* < 0.001), and approximately $1354 more in healthcare costs ($1773 vs $419, *p* < 0.01).

A significantly lower percentage of women with subthreshold psychological symptoms accessed general health-related MBS services compared to the no-CMD group (89.6% vs 97.0%, *p* < 0.01). However, the subthreshold group accessed on average about seven more MBS-funded general health services compared to the no-CMD group (27.1 vs 19.9, *p* < 0.01). Women with a CMD used about five more general health services than women with no-CMD (24.9 vs 19.9, *p* < 0.05). In particular, women with anxiety disorder used about six more services than women with no-CMD (25.8 vs 19.9, *p* < 0.05).

In terms of combined MBS services, women with subthreshold psychological symptoms were less likely to access MBS services than the no-CMD group (89.6% vs 97.0%, *p* < 0.01), but those who accessed MBS services used on average seven more services (27.6 vs 20.0, *p* < 0.01). Women with CMD used about seven more services than women with no-CMD (26.9 vs 20.0, *p* < 0.001). Of women with a CMD, women with a single disorder used five more services than women with no-CMD (25.3 vs 20.0, *p* < 0.05), while multiple-MD used almost 10 more services than no-CMD (29.9 vs 20.0, *p* < 0.01). A statistically significant difference was also found in the number of services used by women with only anxiety diagnoses compared with no-CMD (26.1 vs 20.0, *p* < 0.05). Women with CMD incurred approximately $581 more in annual MBS costs than women with no-CMD ($1889 vs $1305, *p* > 0.01). This difference was driven by women with multiple-MD, having almost $900 more in MBS costs than no-CMD.

Compared to no-CMD, significantly more women with CMD or multiple-MD paid OOP costs for MBS mental health services (9.0% CMD, 18.0% multiple-MD vs 1.3% no-CMD, *p* < 0.001; Supplementary Table S1). Significantly more MBS mental health services incurred OOP cost for multiple-MD compared to no-CMD (9.2 vs 3.3, *p* < 0.05). Forty-four per cent of BA services used by the CMD group incurred OOP costs. Significantly more combined MBS services incurred OOP cost for CMD compared to no-CMD (10.5 vs 7.9, *p* < 0.01).

### Medication use and costs to PBS

**Figure 3.**
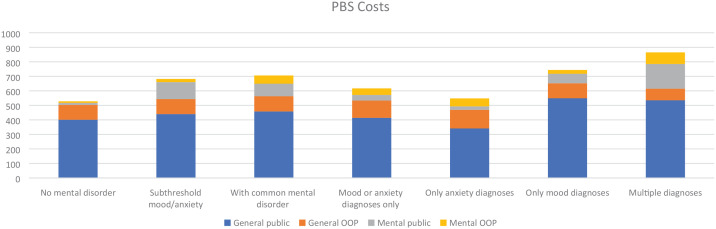
Average annual PBS cost by mental disorder classification. PBS: Pharmaceutical Benefits Scheme; OOP: out-of-pocket. ^a^Includes participants with a combination of at least two categories of diagnosis from anxiety, mood, and other (comorbid eating or substance use disorders).

Figure 3 shows the average annual PBS cost by disease category. There were 607 participants (78%) with PBS data. Of these, 187 (31%) did not receive PBS-funded medications and therefore did not incur any PBS costs. The average annual PBS cost for the subthreshold and CMD groups was higher compared with the no-CMD group ($683 subthreshold, $706 CMD vs $528 no-CMD).

Table 2 shows the results of the two-part and hurdle models for PBS service use and costs. Compared to women with no-CMD who used medications, women with subthreshold psychological symptoms received five more PBS-funded mental health prescriptions (11.1 vs 6.5, *p* < 0.05), and incurred $224 more in mental health-related PBS costs ($366 vs $142, *p* < 0.05). The CMD group had a higher percentage utilising PBS medications, greater numbers of prescriptions and higher costs for mental health prescriptions funded under PBS. Women with CMD were two times more likely to receive a PBS-funded mental health medication (45.4% vs 18.5%, *p* < 0.001), received five more PBS-funded mental health prescriptions (11.7 vs 6.5, *p* < 0.001) and incurred $200 more in mental health PBS costs compared to women with no-CMD ($342 vs $142, *p* < 0.01).

**Table 2.** Two-part and hurdle model results for Pharmaceutical Benefits Scheme prescriptions in the year preceding mental health assessment.

Disorder classification	Mental health prescriptions			General health prescriptions			Combined prescriptions		
	% of participants receiving prescriptions % [95% CI]	Annual number of prescriptions for those who received prescriptions *N* [95% CI]	Average annual cost for those who received prescriptions^a^ $ [95% CI]	% of participants receiving prescriptions % [95% CI]	Annual number of prescriptions for those who received prescriptions *N* [95% CI]	Average annual cost for those who received prescriptions^a^ $ [95% CI]	% of participants using health service % [95% CI]	Annual number of prescriptions for those who received prescriptions *N* [95% CI]	Average annual cost for those who received prescriptions^a^ $ [95% CI]
No common mental disorder	18.5 [14.9, 22.1]	6.5 [5.3, 7.7]	142 [89, 195]	65.8 [61.5, 70.1]	20.0 [17.5, 22.4]	756 [583, 930]	67.8 [63.5, 72.1]	20.6 [18.1, 23.0]	740 [588, 893]
Subthreshold symptoms	29.1 [18.1, 40.0]	11.1 [6.9, 15.2]*	366 [86, 646]*	66.3 [54.9, 77.7]	39.2 [27.2, 51.2]***	947 [439, 1456]	67.9 [56.5, 79.3]	43.3 [30.2, 56.4]***	1268 [619, 1917]*
With common mental disorder	45.4 [36.5, 54.2]***	11.7 [9.0, 14.5]***	342 [185, 499]**	68.0 [59.8, 76.1]	28.4 [21.9, 34.9]**	1047 [608, 1486]	74.3 [66.7, 82.0]	35.8 [27.8, 43.8]***	1226 [753, 1698]*
Mood or anxiety diagnoses only	39.5 [28.7, 50.3]***	10.4 [7.2, 13.6]**	231 [104, 358]	66.5 [56.3, 76.7]	28.9 [20.8, 37.1]*	1148 [553, 1743]	71.4 [61.6, 81.3]	34.4 [24.9, 43.8]***	1184 [621, 1746]
Only anxiety diagnoses	34.3 [21.1, 47.4]*	9.7 [5.7, 13.7]	234 [63, 404]	72.3 [60.4, 84.2]	23.2 [15.5, 31.0]	918 [375, 1460]	76.2 [64.7, 87.7]	27.9 [18.8, 37.0]	1014 [453, 1576]
Only mood diagnoses	48.8 [30.3, 67.3]***	11.3 [6.2, 16.4]*	228 [41, 414]	55.1 [36.8, 73.5]	42.5 [21.7, 63.3]**	1714 [190, 3237]	62.4 [44.4, 80.3]	48.5 [26.0, 70.9]***	1562 [313, 2811]
Multiple diagnoses	56.1 [41.4, 70.7]***	13.4 [8.9, 17.8]***	495 [181, 808]***	70.7 [57.5, 83.9]	27.7 [18.2, 37.2]	901 [360, 1442]	79.7 [67.8, 91.6]	38.1 [25.6, 50.7]***	1291 [569, 2014]

CI: confidence interval; PBS: Pharmaceutical Benefits Scheme.

a Cost includes the payments made by the Commonwealth government through the PBS and the out-of-pocket cost made by the consumer.

* *p* < 0.05; **p < 0.01; ***p < 0.001.

Compared to women with no-CMD, women with a single CMD were twice as likely to receive a PBS mental health medication (39.5% vs 18.5%, *p* < 0.001) and received over three additional mental health prescriptions (10.4 vs 6.5, *p* < 0.01). On the other hand, multiple-MD were three times more likely to receive a PBS mental health medication (56.1% vs 18.5%, *p* < 0.001), with about seven more mental health prescriptions (13.4 vs 6.5, *p* < 0.001), and incurred $352 more in PBS mental health costs ($495 vs $142, *p* < 0.001).

In analysis of specific disorders, women with anxiety disorder, when compared to no-CMD, were almost twice as likely to receive a PBS mental health medication (34.3% vs 18.5%, *p* < 0.05). Women with mood disorder were over two and a half times more likely to receive a PBS mental health medication (48.8% vs 18.5%, *p* < 0.001) and received about five more PBS mental health prescriptions (11.3 vs 6.5, *p* < 0.05).

Women in the subthreshold group received significantly more general PBS prescriptions compared to the no-CMD group (39.2 vs 20.0, *p* < 0.001). Women with CMD also received significantly more general PBS prescriptions compared to the no-CMD group (28.4 vs 20.0, *p* < 0.01). Women with a single CMD or a mood disorder received significantly more PBS general prescriptions compared with no-CMD (single CMD 28.9, *p* < 0.05; mood disorder 42.5 vs 20.0 no-CMD, *p* < 0.01).

Compared to women with no-CMD, subthreshold (43.3 vs 20.6, *p* < 0.001), CMD (35.8 vs 20.6, *p* < 0.001), single CMD (34.4 vs 20.6, *p* < 0.001), multiple-MD (38.1 vs 20.6, *p* < 0.001) and mood disorder (48.5 vs 20.6, *p* < 0.001) received significantly higher numbers of combined mental and general PBS prescriptions. For PBS costs, differences were only found in subthreshold ($1268 vs $740, *p* < 0.05) and CMD groups ($1226 vs $740, *p* < 0.05) when compared with no-CMD.

Compared to no-CMD, significantly more women with subthreshold psychological symptoms utilised PBS mental health medications incurring OOP costs (28.8% vs 16.9%, *p* < 0.05; Supplementary Table S2). More women with CMD, a single CMD, mood disorder or multiple-MD utilised PBS mental health medications incurring OOP costs than no-CMD (44.9% any CMD, 39.0% single CMD, 48.5% mood disorder, 55.6% multiple-MD vs 16.9% no-CMD, *p* < 0.001). Significantly more combined prescriptions attracted OOP cost for women with subthreshold psychological symptoms or CMD compared to no-CMD (28.9 subthreshold, 28.7 CMD vs 17.1 no-CMD, *p* < 0.001) leading to significantly higher annual OOP costs for CMD ($249 vs $162, *p* < 0.001).

## Discussion

To our knowledge, this is the first study to present medical service and medication costs using MBS and PBS data for women residing in Australia with subthreshold symptoms or clinically diagnosed CMD. This analysis found women experiencing psychological symptoms but not reaching thresholds for diagnosis were more likely to access MBS mental health services relative to women without a CMD diagnosis or symptoms. They also had a lower percentage accessing general health services funded through MBS, but those accessing services utilised significantly more services. These differences did not translate into statistically significant differences in MBS costs. The subthreshold group received more PBS mental and general prescriptions and had higher total PBS costs compared to women without CMDs. These results contrast with Le et al. (2021) that found young people (ages 4–17 years) classified as subthreshold accessed significantly more MBS and PBS services for mental and general health but the only significant cost difference was found for MBS mental health services. Further research on the relationship between mental health symptoms, healthcare service use and cost in adults is needed.

For women with CMDs, 19.7% received at least one mental health-related medical service through an MBS reimbursed provider over the preceding year. The average annual cost for MBS mental health services accessed was $234. This is considerably lower than the 40.7% of women self-reporting healthcare professional consultations for their mental health (Burgess et al., 2009), and the $482 average health sector cost of these consults from people with a high prevalence mental health diagnosis (depression, anxiety or substance use) reported from the 2007 NSMHWB (Lee et al., 2017). These differences are likely due to the inclusion of hospital admissions in the NSMHWB analyses, since MBS data do not include public hospital admission costs.

A significantly higher percentage of women with CMD were paying OOP costs for MBS mental health services, attributable to private psychiatrist visits and BA services. The 44% of BA items attracting OOP costs in our study are comparable to the 36–47% of BA services with OOP costs between 2018 and 2021 (Prikis et al., 2022). When combined with the significantly higher average annual OOP costs women with CMD are paying for prescription medications, this presents a considerable barrier to accessing mental and general healthcare.

The women with diagnosed CMDs had more PBS prescription claims, and higher medication costs compared to women without CMDs. We found 45% of women with a CMD received psychotropic medications under the PBS, with an average cost of $342. The proportion using psychotropic medications in the current study is higher than the 23% of adults reporting the use of prescription medications for their mental health from the 2007 NSMHWB (Lee et al., 2017). This may be due to this study’s focus on women while Lee et al. included both males and females. Women have a higher prevalence of depressive, anxiety and eating disorders as well as being more likely to access mental health services than men (Australian Bureau of Statistics, 2008; Ferrari et al., 2022). The average cost of medications was higher than the $238 reported from the 2007 NSMHWB for people with high-prevalence MDs possibly due to the use of newer medications covered by patent protection.

Women with CMDs utilised significantly more general health MBS and PBS services compared to women without a CMD diagnosis or symptoms. However, this additional service use did not translate into significantly greater costs. A previous study using a US national survey found the direct cost of treating comorbid conditions was approximately three times higher than the direct cost attributed to treating major depressive disorder (MDD), mostly attributable to the treatment of pain (Greenberg et al., 2015).

Women with multiple CMDs utilised the highest number of MBS services and had the highest MBS cost; they received the second highest number of PBS prescriptions and had PBS costs similar to the subthreshold group. Our results highlight the burden associated with multiple CMDs found in previous research (Christensen et al., 2023; Lee et al., 2017; Teesson et al., 2009).

### Strengths and limitations

The population-based sampling method used to recruit GOS participants provides results generalisable to the Geelong region. Furthermore, the use of a gold-standard semi-structured clinical interview provided accuracy in diagnosis. The administrative claims from MBS and PBS provided accurate recording of subsidised services and costs incurred in Australia under these Commonwealth systems.

However, the MBS and PBS costs reported here are an underestimate of the cost to manage mental health. The cost of public hospital admissions, emergency department visits and community-based mental health services provided through state-funded services are not provided through these data. The limitations of MBS and PBS claims data prevented detailed costing of non-mental health services and medications for people with MDs within this study.

The analyses were limited to participants providing access to their MBS and PBS data, and did not use imputation of missing data. A further limitation is the lack of sensitivity analysis to exclude costs incurred during private hospital admissions, resulting from omission of an indicator within the MBS data extraction.

The classification of MBS services within this study was less than optimal due to a lack of diagnosis with each claim. We acknowledge there may have been some services relating to CMD or possibly had a mental health component that were not captured in the mental health-related class. GP services, in particular, can involve discussions around mental health but would ultimately be billed under generic GP service items. In a previous study, it was estimated that around 2% of GP consultations were mental health specific while 12% were mental health related (Britt et al., 2016).

Finally, while quantifying the cost associated with CMD and symptoms is important to describe economic burden, this information alone does not indicate whether investment in an area is cost-effective.

## Conclusion

This analysis demonstrates that CMDs and psychological symptoms continue to place a substantial economic burden on Australian Commonwealth-funded healthcare services funded under the MBS and PBS systems. Additional research to understand the impact of psychological symptoms without a clinical diagnosis is needed.

## Supplemental Material

sj-docx-1-anp-10.1177_00048674241229931 – Supplemental material for Health service and medication costs associated with common mental disorders and subthreshold symptoms in women: Findings from the Geelong Osteoporosis Study in Australia Supplemental material, sj-docx-1-anp-10.1177_00048674241229931 for Health service and medication costs associated with common mental disorders and subthreshold symptoms in women: Findings from the Geelong Osteoporosis Study in Australia by Mary Lou Chatterton, Jan Faller, Long Khanh-Dao Le, Lidia Engel, Lana J Williams, Julie A Pasco and Cathy Mihalopoulos in Australian & New Zealand Journal of Psychiatry
