# Supplementary material for: Health service and medication costs associated with common mental disorders and subthreshold symptoms in women: Findings from the Geelong Osteoporosis Study in Australia
Source: Aust N Z J Psychiatry. 2024 Feb 11;58(5):404–15. doi: 10.1177/00048674241229931 (PMC11055409; doi:10.1177/00048674241229931)
Supplement: sj-docx-1-anp-10.1177_00048674241229931 – Supplemental material for Health service and medication costs associated with common mental disorders and subthreshold symptoms in women: Findings from the Geelong Osteoporosis Study in Australia [file sj-docx-1-anp-10.1177_00048674241229931.docx]

**Supplementary Materials**

**Table S1. Two-part and hurdle model results for out of pocket (OOP) payments on Medicare Benefits Schedule services in the year preceding mental health assessment**

|  |  | **Mental health services** | | |  | **General health services** | | |  | **Combined services** | | |
| --- | --- | --- | --- | --- | --- | --- | --- | --- | --- | --- | --- | --- |
| **Disorder classification** |  | **% of participants paying OOP for health services % (95% CI)** | **Annual number of services with OOP for those who paid OOP**  **N (95% CI)** | **Average annual OOP for those who paid OOP**  **$ (95% CI)** |  | **% of participants paying OOP for health services**  **% (95% CI)** | **Annual number of services with OOP for those who paid OOP N (95% CI)** | **Average annual OOP for those who paid OOP**  **$ (95% CI)** |  | **% of participants paying OOP for health services**  **% (95% CI)** | **Annual number of services with OOP for those who paid OOP N (95% CI)** | **Average annual OOP for those who paid OOP**  **$ (95% CI)** |
|  | | | | | | | | | | | | |
| **No common mental disorder** |  | 1.3 (0.2, 2.3) | 3.3 (0.2, 6.5) | 279 (0, 759) |  | 82.3 (78.9, 85.8) | 7.8 (7.1, 8.6) | 423 (346, 499) |  | 82.4 (78.9, 85.8) | 7.9 (7.1, 8.6) | 426 (349, 502) |
| **Subthreshold symptoms** |  | 2.4 (0, 5.8) | 7.3 (0, 14.7) | 249 (0, 649) |  | 79.1 (69.6, 88.6) | 9.0 (6.7, 11.3) | 472 (245, 698) |  | 79.1 (69.6, 88.6) | 9.2 (6.9, 11.6) | 483 (254, 713) |
| **With common mental disorder** |  | 9.0 (4.4, 13.5)*** | 7.4 (4.5, 10.3) | 397 (117, 677) |  | 83.7 (77.3, 90.1) | 9.7 (7.9, 11.5)* | 491 (320, 662) |  | 85.1 (79.0, 91.3) | 10.5 (8.6, 12.4)** | 531 (350, 713) |
| **Mood or anxiety diagnoses only** |  | 4.3 (0.3, 8.3) | 3.5 (0.2, 6.8) | 146 (0, 383) |  | 87.5 (80.4, 94.6) | 11.0 (8.6, 13.4)** | 570 (332, 807) |  | 88.7 (81.9, 95.5) | 11.0 (8.6, 13.4)** | 568 (334, 803) |
| **Only anxiety diagnoses** |  | 3.6 (0, 8.3) | 3.6 (0, 8.2) | 196 (0, 630) |  | 91.5 (84.2, 98.9) | 11.2 (8.2, 14.2)* | 569 (281, 857) |  | 93.4 (86.8, 99.9)* | 11.0 (8.1, 13.9)* | 558 (279, 837) |
| **Only mood diagnoses** |  | 5.5 (0, 13.3) | 3.4 (0, 7.5) | 113 (0, 335) |  | 79.6 (64.7, 94.5) | 10.4 (6.3, 14.5) | 572 (142, 1001) |  | 79.5 (64.6, 94.4) | 10.8 (6.6, 15.1) | 590 (148, 1032) |
| **Multiple diagnoses** |  | 18.0 (7.3, 28.7)*** | 9.2 (4.7, 13.6)* | 478 (94, 862) |  | 76.6 (64.3, 88.9) | 6.9 (4.6, 9.2) | 323 (127, 520) |  | 78.7 (66.9, 90.6) | 9.5 (6.4, 12.6) | 452 (178, 726) |

* p<0.05; ** p<0.01; *** p<0.001

**Table S2. Two-part and hurdle model results for Out of Pocket (OOP) payments on Pharmaceutical Benefits Scheme prescriptions in the year preceding mental health assessment**

|  |  | | **Mental health prescriptions** | | |  | **General health prescriptions** | | |  | **Combined prescriptions** | | |
| --- | --- | --- | --- | --- | --- | --- | --- | --- | --- | --- | --- | --- | --- |
| **Disorder classification** | |  | **% of participants paying OOP for prescriptions % (95% CI)** | **Annual number of prescriptions with OOP for those who paid OOP**  **N (95% CI)** | **Average annual OOP for those who paid OOP**  **$ (95% CI)** |  | **% of participants paying OOP for prescriptions % (95% CI)** | **Annual number of prescriptions with OOP for those who paid OOP**  **N (95% CI)** | **Average annual OOP for those who paid OOP $ (95% CI)** |  | **% of participants paying OOP for prescriptions % (95% CI)** | **Annual number of prescriptions with OOP for those who paid OOP**  **N (95% CI)** | **Average annual OOP for those who paid OOP**  **$ (95% CI)** |
|  | | | | | | | | | | | | | |
| **No common mental disorder** | |  | 16.9 (13.4, 20.4) | 6.1 (4.9, 7.2) | 66 (46, 87) |  | 65.6 (61.3, 69.9) | 16.6 (14.7, 18.5) | 154 (133, 174) |  | 67.6 (63.3, 71.9) | 17.1 (15.2, 19.1) | 162 (142, 182) |
| **Subthreshold symptoms** | |  | 28.8 (17.9, 39.8)* | 8.5 (5.4, 11.5) | 68 (30, 106) |  | 66.3 (54.8, 77.7) | 25.6 (18.3, 32.8)** | 171 (112, 230) |  | 67.8 (56.5, 79.2) | 28.9 (20.8, 37.1)*** | 200 (135, 264) |
| **With common mental disorder** | |  | 44.9 (36.0, 53.8)*** | 10.2 (7.9, 12.5)*** | 128 (83, 174)** |  | 67.9 (59.7, 76.0) | 22.5 (17.7, 27.2)** | 170 (127, 213) |  | 74.3 (66.6, 82.0) | 28.7 (22.8, 34.6)*** | 249 (190, 308)*** |
| **Mood or anxiety diagnoses only** | |  | 39.0 (28.2, 49.8)*** | 9.2 (6.5, 11.9)* | 110 (59, 160) |  | 66.4 (56.2, 76.6) | 24.2 (17.9, 30.5)** | 198 (137, 259) |  | 71.4 (61.5, 81.2) | 28.8 (21.4, 36.1)*** | 255 (181, 330)** |
| **Only anxiety diagnoses** | |  | 33.7 (20.5, 46.8)** | 9.2 (5.6, 12.8) | 146 (58, 233)* |  | 72.2 (60.3, 84.2) | 20.8 (14.4, 27.1) | 200 (126, 274) |  | 76.1 (64.6, 87.6) | 24.7 (17.2, 32.1)* | 275 (179, 371)** |
| **Only mood diagnoses** | |  | 48.5 (30.0, 67.1)*** | 9.3 (5.2, 13.3) | 65 (21, 109) |  | 55.1 (36.7, 73.5) | 32.6 (17.7, 47.5)** | 193 (84, 302) |  | 62.4 (44.3, 80.4) | 37.9 (21.4, 54.4)*** | 213 (104, 323) |
| **Multiple diagnoses** | |  | 55.6 (40.8, 70.4)*** | 11.4 (7.8, 15.1)*** | 149 (72, 227)** |  | 70.6 (57.3, 83.8) | 19.8 (13.4, 26.2) | 125 (76, 174) |  | 79.6 (67.7, 91.6) | 28.8 (19.9, 37.7)** | 238 (150, 325) |

* p<0.05; ** p<0.01; *** p<0.001
